# Abstracts and Gleanings

**Published:** 1887-04

**Authors:** 


### ABSTRACTS AND GLEANINGS.

**Rest for Painful Eyes Not Always Effectual.**—Dr. Chisolm in *Medical and Surgical Reporter*, says: How many thousands in this country to day are impatiently and uselessly resting eyes that pain when put to near work, when a pair of properly adjusted spectacles will correct the evil?

Nearly every day I restore some restless patient to his work who had sought in vain relief from eye pains in rest; or I assist some ambitious person, who having acquired an enviable start in life, feels that his painful eyes have become barriers to further study and prospective promotion. Daily, by the use of properly selected glasses, I cure headaches, often of years' duration, and which have resisted every species of medication. In so doing I have often been able to satisfy anxious patients that their brains, stomachs, livers, kidneys, or uteri, have been accused wrongfully of producing the headaches, and that these have ever been innocent and healthy organs. The following remarks I have frequently heard from patients to whom I had recently prescribed astigmatic glasses: "For one week, ever since I put on the spectacles, I have been free from headache, and it is a freedom that I have not had before for years."

Although most astigmatic eyes cause headache and eye pains, if the eyes are much used in fine work, especially by artificial light, I find cases of faulty refraction from astigmatism in which headache is not and has never been an annoying symptom.

In some astigmatic persons a strong muscular development enables them to conceal the corneal irregularity. Should any disturbance of the system temporarily weaken this muscular power, the eye muscles, along with the other muscles of the body, are weakened and unable to keep up their work; then are pains induced. If it be a bilious or gastric disturbance, its temporary influence over the muscles is mistaken for the actual cause of the headache, when it is only the indirect cause, permitting the latent trouble to become manifest. If the astigmatism did not exist in a concealed form, there would be no headache on use of the eyes barring these general disturbances.

Again, in nervous persons, especially in females, I have found great suffering about the head and eyes, clearly traceable to a small degree of irregular refraction, and promptly corrected by the constant use of carefully adjusted cylinder lenses.

The case with a report of which I will close this paper is one of unusual severity in effects, although a high degree of astigmatism did not exist. Such extreme discomfort as this lady suffered is fortunately not often found. The case is also peculiar from the length of time that she suffered before her eyes were suspected of being the source of the trouble. In this age of diffusion of medical knowledge by means of many medical journals, physicians are on the alert to distinguish eye headaches from the headaches caused by other organic disturbances, and usually at an early day invoke the aid of the specialist in eye diseases to remedy the evil. In her own case several years elapsed in testing newspaper remedies for headache, having lost faith in physicians from her earlier medical experiences. The case, however, will illustrate the efficacy of proper glasses in relieving even years of suffering.

Mrs. F., aged 38, the mother of several children, has been a martyr to headaches since childhood; and during the past thirteen years, since her married life, has been often nearly crazy from them. Any close eye work, continued for even a short time, would send her to bed with a raging headache. On an average she has spent one day out of every week in a dark room, and that has been kept up for months at a time. If she felt bright and applied herself to complete any needle-work, so necessary with a growing family, she never failed to pay the penalty in severe head and eye suffering. When she came first to my office, she frankly told me that she had come because she had been advised, not that she expected any benefit, for she had no faith in any curative agent whatever, having years since exhausted them all without finding any relief. She gave me this very clear history of her case: "Dr. A. has always been my family physician, and in him I have every confidence. Having in my early married life exhausted his skill in vain attempts at relieving me of suffering, he gave up treating me for these headaches many years ago. Under his advice I had consulted Prof. B.; you know him to be one of our leading practitioners. He acknowledged that I had a good family doctor, but thought that something might have been overlooked, and he hoped to find me a remedy. He varied his medicines, as one after another failed to procure me relief, and finally he advised a visit to the seashore. I spent six weeks at Cape May, and while there rested my eyes from all work, eschewing both reading and sewing. I returned home with body invigorated by the salt baths, and was free from pain. As soon as I commenced using my eyes in sewing all the old distressing symptoms returned. My family physician and friend, seeing me in some of these terrible attacks, advised me to consult another physician, Prof. C., who you know has the reputation of a skillful physician. He had me under his professional care all winter and spring. Summer found me no better. Any use of the eyes in sewing or reading sent me to bed with twenty-four hours of suffering before me. He finally advised a course of mineral waters, and sent me to the White Sulphur Springs of Virginia. There I spent two months, which improved me much in health. In the fall I returned to Baltimore, looking and feeling well A very few days of housekeeping showed me that the long rest at the springs and the drinking of sulphur waters had brought me no permanent good. My head at times ached as badly as ever.

"I now despaired of ever getting relief, because I had sought the best medical advice at my command and all to no purpose. Some of my friends in their anxiety to see me cured of the daily suffering, advised me to try Homœopathy. I accepted the suggestion, and sent for Dr. D———. He examined carefully into my case, and said that he could cure me. With these assurances from the new physician, my feeling barometer at once went up, and my future prospects brightened. I entered actively into the course of medication mapped out by him. I took his mixtures hour by hour, for days and weeks, my faith growing unfortunately less with the monotony of the dosing. Finally, as my headaches were not mitigated even by the long continued treatment, I gave up all hope, and dismissed the Homœopathic physician.

"I felt that my case was now beyond medical cure, and I became despondent and rash. In my anxiety to secure relief I have tried anything that any one would suggest. I believe that during the last six years I have taken every quack remedy warranted to cure headaches that I could hear of, as published in the newspapers, and my many friends have kept me well supplied with this kind of information. Recently I have heard how Miss E——— has been cured of constant headaches by wearing glasses, and my friends have suggested that I have my eyes examined. On the principle that, in my desire to escape this bodily torment, I have been willing to try every treatment that has been brought to my notice, I have come to have you examine my painful eyes; but I must tell you candidly that I expect no benefit and have given up all hope of obtaining relief."

Upon examination, I found that she could read the finest print, but only for a few lines. Her distant vision was also acute. Fixing the eyes upon the clock dial trial card for a short time caused pain in the head and eyes, and also induced a feeling of nausea. I found that she could clearly see the vertical lines of the test card, but dimly those which were horizontally placed. I selected from the trial case a magnifying lens which would make these blurred lines perfectly clear for each eye, and finding the corresponding cylinders, adjusted them at the proper angle in a trial frame. These I placed before her eyes. To her surprise, not only did all the lines come out with equal boldness of color and of definition, but she found herself able to stare at them without inconvenience. After she had worn the glasses for some minutes, feeling great comfort from them, I removed the frames, when immediately the nausea previously experienced came on. The restoration of the glasses brought back strength of vision and comfort. I prescribed for her the proper cylinder lenses set at an angle of 180° in spectacle frames, to be constantly worn. So anxious was she to test these spectacles that on her way home from my office she called at the optician's and remained in the store while the glasses were being fitted to the frames which she had selected. When they were ready she put them on at once and sallied forth. Before getting home she found herself walking with a degree of comfort which she had not known for months.

The rapid improvement commenced from that hour. Her headache disappeared within three weeks by the rest which her eyes enjoyed from the constant wearing of the spectacles. Now she makes her eyes do just what she pleases. Her constant headaches are bygones, and are only remembered from the years of torture through which she had passed. Her face has become free from care as her head is free from pains. Her relief by such apparently simple means, and without medicines, is called a miracle by herself, and is a marvel to her friends. No amount of rest without these cylinder glasses could have effected this cure from suffering. It had been thoroughly tested, and had been found as useless as the many years her body had been drugged. *Cylinder glasses alone could, and have cured her.*

**Salicylate of Ammonium.**—Dr. J. R. Barnett, of Neenah, Wis., (in Journal of the American Association) says:

The salicylate of ammonium is to be ranked among the most efficient of the antipyretics.

As an antipyretic in all fevers characterized by extreme adynamia it ranks among the safest, owing to its ammonia base.

It is stimulant as well as antipyretic, and thus of itself fulfils indications otherwise only met by a combination of remedies.

It is an agent of wide germicidal powers, being promptly efficient in affections of great etiological and pathological differences, each confessedly arising from its own proper specific infecting micro-organism.

As a remedial agent in typhoid and remittent fevers it is unsurpassed, absorbing them at the outset, under favorable conditions, and greatly mitigating their severity and danger under circumstances less favorable.

It is entitled to confidence in the treatment of pulmonary inflammations, either idiopathic or septic, and probably eliminates the dangerous factor, embolism, with greater certainty than any other prime curative agent.

It is the most efficient known remedy in puerperal septicemia, and probably also in most septic inflammations of non-puerperal origin.

It is worthy of a trial in non-tubercular cerebral meningitis, as it gives some promise of relief in an affection which has hitherto resisted, if not resented all modes of medical treatment.

Since the submission of the foregoing report the clinical material at my disposal has enabled me to fortify its conclusions by additional data.

The case of cerebral meningitis referred to recovered completely, partial deafness, however, continuing several weeks.

Another case convalesced at end of second week, the symptoms during four or five days denoting great danger. Among the earlier of these were repeated convulsions. In this case the ice-cap could not be employed, and evaporating lotions had to be used instead; which, it seemed to me, could have afforded but little help to the constitutional treatment, either in the control of the cerebral circulation or the high temperature.

Some twenty-five or more cases of typhoid or allied fevers have been under my care during the present autumn. At least this number, had they been left uninfluenced by medical treatment, would have pursued, with substantial fidelity to the clinical history of these fevers, the usual course.

With one exception, they were subjected to the salicylate of ammonia treatment only, and uniformly without alcoholic stimulation, and with the exception noted they all recovered. Of these cases all were convalescing by the close of the second week. To speak more definitely, three showed entire absence of fever on the twelfth day, three more on the ninth ; all the rest were convalescent at the end of the first week, or earlier.

The case in which the ammonium treatment was not strictly adhered to died on the ninth day of the fever, two days after the occurrence of an enormous nasal hemorrhage. Epistaxis had occurred several times before I saw the case, and it was an almost daily phenomenon throughout. Thinking that the salicylate of ammonia might contribute in some degree to the existing hypinosis, I substituted quinine for it, without, however, improving the condition in the least. The prognosis was extremely favorable up to the day of the severe hemorrhage. After this the decline was phenomenal in its rapidity.

In the cases recovering after but few days of fever there was, not seldom, a tendency to relapse or return of high temperature, where the antipyretic was prematurely withdrawn. It seemed analagous to the recrudescence of rheumatism frequently observed in the too early abandonment of salicylic acid. I soon learned from them the necessity of continuing the remedy in reduced quantity and at lengthening intervals during several days of normal temperature. With this precaution, when the fever leaves it ends definitively.

**Anæsthetics.** — In a discussion on "Is Bromide of Ethyl as dangerous to life as Chloroform ?" by the Philadelphia County Medical Society, Dr. William H. Pancoast said :

I have had very little experience with bromide of ethyl, having used it but a few times. From my own experience, I would say that where an operation requires any length of time ether is the most effective anæsthetic. My plan is to give the ether without allowing the patient to be held, and if the patient becomes excited, to throw on the towel about a teaspoonful of chloroform, and after the stage of excitement has passed to continue the use of ether. In office practice I use occasionally chloroform in slight operations, or nitric oxide gas. When I employ chloroform I direct the patient to hold up his hands, and the moment he begins to feel the effect of the anæsthetic the hands will drop, and at that moment any slight operation may be performed. I am satisfied that when ether is given, patients are often ether-logged. In many cases where death is attributed to shock, I think it is due to the excessive use of ether. Ether and chloroform should not be mixed. The mixture of ether, chloroform, and alcohol is very dangerous. With such a mixture, you are giving chloroform without the precautions which chloroform requires. I have given ether in cases of phthisis and in cardiac disease, and, in the last, have found the heart become steadier under its proper administration.

Dr. W. Joseph Hearn : I have not had a large experience in the use of bromide of ethyl, but I believe that it must be watched as closely as chloroform. I believe that, by the careful and gradual inhalation of chloroform, every case can be anæsthetized safely.

In private practice I frequently use chloroform. I believe that there is less shock from its use than from ether. In cases where there has been great shock I have used chloroform with satisfactory results. Where a person is much debilitated, it does not require much of any anæsthetic to produce anæsthesia. I think we can make no comparison between the effects of an anæsthetic on animals and on man. I have frequently given chloroform to dogs, although it has invariably resulted in death.

Dr. Laurence Turnbull: With the utmost care, dogs may be put under the influence of chloroform, but if we are careless, in an instant the dog is dead. The late Dr. Griswold recently stated that ethyl bromide would kill dogs quicker than chloroform. This is not the case, as the observations in my paper will show. Dr. John B. Roberts has reported four deaths from the use of ether, where the fatal result was due to engorgement of the bronchii and paralysis. Ether is frequently continued much longer and in larger quantity in the lungs than there is any necessity for. This is especially true in regard to Squibb's ether, which contains very little water. In using this, it is well to moisten the sponge with warm water before applying the ether—*Medical Reporter.*

**A Suture that is Painless and Leaves No Scar.**—Very frequently after the healing of wounds of the face the most noticeable features of the scar are the pits and creases caused by the sutures used in approximating and closing the wound. In many, indeed in the great majority of the cases of incised wounds of the face and other portions of the body where the treatment is applicable, the ordinary methods of stitching may be replaced by the following, which not only avoids pitting and marking, but has the great advantage of being painless:

Cut two pieces of adhesive plaster somewhat longer than the wound, and from an inch and a quarter to an inch-and-a-half wide. They should be shaped so that one edge of each will follow the course of the lesion, if the latter be straight or simply curved; but if the wound be irregular or "zig-zagged" it is better to use more pieces. Turn the inner edge (or that intended to be next the wound) of each of these strips under, so as to form a non-adhesive border a quarter of an inch wide, and leave an adhesive surface of from three-quarters of an inch to one inch in width. Apply these to the uninjured skin on each side of the wound, and make them adhere firmly by holding them to this with a hot, dry towel. The stitches may now be taken from side to side, thrusting the needle through the doubled edge of the plaster instead of through the skin, and after the fashion of a shoe-lacing, uninterrupted. By drawing on the ends of the thread the nicest coaptation of the skin may be obtained. Instead of the ordinary plaster a perforated or porous plaster may be used, the perforations being utilized as eyelets.

The writer devised and used this method of suturing ten or twelve years ago in the treatment (in connection with Dr. H. C. Dunavant, of Osceola, Ark.,) of a knife slash across the face of a little boy. He afterwards used it several times with the best results, but had forgotten it until reminded of it by reading M. Marc See's new method of quilled suturing, given elsewhere in this journal.—*St. Louis Medical and Surgical Journal.*

**The Treatment of Renal Calculus.**—Dr. James Tyson, in *Boston Medical and Surgical Journal:* The correct treatment for uric acid is the alkaline treatment. For this purpose any alkali may be used, such as citrate or acetate of potassium. I prefer the citrate of potassium, in twenty-grain doses, four times a day, each time in eight ounces of water. Dilution is very important. It is of the greatest importance that you should, as it were, wash the *debris* of tissue metamorphosis. The introduction of large quantities of water into the system is an important element in the treatment. It is of the good effects of such treatment that the reputation of many of the mineral springs in the treatment of gravel is due.

The same plan applies to the oxalate-of-lime calculus, but with less prospect of success, so far as solution of the stone is concerned. You cannot dissolve the oxalate of lime with acids or alkalies of such strength as it would be proper to use in the body. At the same time, the methods which prevent the formation of uric acid will prevent the formation of oxalate of lime. In the treatment of both of these varieties diet is of the greatest importance. In neither of them should the patient be allowed to eat a large amount of nitrogenous food, as meat or eggs. Fresh, succulent vegetables may be allowed in some cases, with a moderate amount of meat, or in bad cases, no meat at all. If there is one condition in which the good reputation of a pure milk diet is justified, it is the treatment of uric-acid culculus. So often have I been successful in obstinate cases of frequently recurring uric-acid gravel with the pure skim-milk treatment, that it is the first thing that I offer the patient. I have noticed that if the skim-milk diet is kept up for a month or six weeks, and then stopped, the attacks of colic do not recur. In carrying out this plan of treatment, I recommend that during the first day a glass of milk be taken every two hours. After this, the quantity is gradually increased until sufficient to satisfy the patient is taken. The quantity required to sustain life varies between three and seven quarts per day. The milk acts both as a diluent and as an alkaline fluid.

If the stone is composed of phosphates, the milk diet is much less valuable as a cure. The indication is here to increase the acidity of the urine; but while it is true that the phosphates are soluble in acids, it is, practically speaking, impossible to give acids in amount sufficient to affect the calculus without injuring the system. By the use of benzoic acid, in 10-grain doses, the reaction of the urine may be rendered acid, and the further deposition of phosphates may be thus prevented, but nothing can be done towards dissolving the stone.—*Epitome.*

**The Treatment of Compound Fractures.**—Dr. Frederick S. Dennis, says in *Medical News*, November 13, 1886: In order to appreciate what progress surgery has made during the last half century in the treatment of compound fractures, it is interesting to examine hospital reports embracing this period.

If now some statistics are examined after the introduction of antiseptics, with a view to ascertain their influence upon the rate of mortality, one is at once confronted with an astonishing fact. If, for example, Billroth's table is referred to after the use of antiseptics, the death-rate is reduced to one-tenth of what it had been formerly in the treatment of compound fractures. The influence, therefore, of antiseptics has caused the death-rate to fall from twenty to sixty per cent. to about four per cent. within a period of a few years.

In the present report of 516 cases of compound fractures, if we compare the fractures of the extremities only, as has been the case in the above tables, there is no death from septopyæmia, and thus the rate of mortality from blood-poisoning is now reduced from sixty per cent. to a figure which is represented by a cipher. It may be said, therefore, that pyæmia and septicæmia, which destroyed as many as sixty per cent. of human lives in cases of compound fractures, have been practically eliminated as causes of death.

**The Removal of Superfluous Hair.**—Dr. Julia W. Carpenter, in *Cincinnati College of Medicine*, says: Among the main remedies for such cases glacial acetic acid has some qualities that highly recommend it.

First, its caustic properties are superficial, it does not sink into the tissues, and thus the depth to which one wishes to penetrate can be easily controlled.

Second, it is painless when applied with proper care, the most that it causes being a little itching or very little smarting after the skin is broken.

Third, there seems to be no necessity for a resulting scar. In the many cases in which I have used it there was not left a mark of any kind, except in two, where, in spite of warning, the patient could not resist the temptation to pick at and pull off the little scab as it formed. A slight depression was the result.

A little pine stick, sharpened to a very fine point, is dipped in the glacial acetic acid and applied to the skin by the side of one hair, this hair being put slightly on the stretch, either with the fingers or fine pincers. The skin after touching it several times with an interval of a few moments between. is soon softened, so that the point can enter the hair follicle. The hair then comes out easily and another application is made, letting the point pass as deeply into the follicle as it will.

Sometimes I have used the head of a needle for the last application, the eye of the needle holding the acid, which is thus carried to the bottom of the follicle.

About ten hairs were removed at a sitting, and when the work was completed the face presented a very different aspect minus its appendages.

Of course in removing many hairs close together there is some danger of changing the natural appearance of the integument from this fact. In destroying the hair follicle the sebaceous cyst is also often destroyed, and if many of these are wanting it gives a dry or even glazed appearance to the skin. This result follows no more from this method, however, than from any other, as electrolysis.

When the growth is fine and close together, electrolysis would be a better way; but when not too close, or in cases of isolated hairs, this means is very simple and efficient.

**Surgical Infection.**—Dr. Geo. R. Fowler, (*New York Medical Journal*, November 20, 1886) in the course of a paper on the above subject says: I am not an advocate of the Lister dressing. I believe it to be cumbrous, expensive, and open to other objections as well. I believe in the application of a dressing which will allow of the free entrance of air, and thus favor the rapid desiccation of the discharges. The moss dressings of the Germans or the paper-wool dressing introduced by myself fulfills all the requirements of dry wound dressing perfectly. Of course, this is only to be applied after all so-called "dead spaces" have been provided against by drainage or suturing, and the wound has been sterilized and closed. But that Lister's or any other dressing of an antiseptic nature prevents us from knowing what is going on in the wound is a fallacy. Baron Larrey the elder, the great master of French surgery, as well as the equally clear-headed and successful English teacher of our art, Sir Astley Cooper in their day declared against meddlesome surgery; and the reasons given for this are as cogent now as then. Failure of drainage, sepsis, and other untoward conditions are quickly announced by the thermometer or by the occurrence of pain. The time occupied and material used in an antiseptic dressing are trival matters compared to the absence of necessity of frequent changes, to say nothing of the surgeon's peace of mind when he feels the assurance that all will go well with the wound as well as the patient, and that if failure occur it is through no fault or neglect of his own. The requisites in these days of simplicity in dressing are few and inexpensive. Mercuric bichloride, or even common salt, or diluted vinegar, if nothing better is at hand, is easily obtainable, and sawdust, or absorbent paper torn into narrow strips and made into a cushion, constituting the before-mentioned paper-wool, is all that is really needful, in addition to what good surgery always requires —namely, cleanliness and proper measures for closing the wound and draining it; or, these not being deemed needful in the particular case under notice, support and compression.—*Epitome.*

**Antipyrine in Pneumonia.**—Dr. Jesse B. Hall, of Bellaire, Mich., writes to the *Medical Age*: I have used antipyrine in the following cases of pneumonia:

Case 1.—Katie B——, æt eighteen months. Broncho-pneumonia. Temperature, 103°; pulse, 135; respiration, 50 per minute; coughing quite frequently and very deep. Administered antipyrine, grains 4, every three hours. Improvement from first dose.

Case 2.—Louis H——, æt three years. Congestion and slight inflammation of the lungs. Temperature, 102°; pulse, 125; respiration, 48. Administered antipyrine, grains 4, every three hours. Rapid recovery.

Case 3.—Infant son of Patrick S——, æt two and a half months. Broncho-pneumonia with cerebral congestion. Administered antipyrine, grains 3, every two hours. Head symptoms disappeared in ten hours, and the patient made a rapid recovery.

Case 4.—Marl D——, æt 3 months. Broncho-pneumonia. The attending physician having pronounced the case hopeless. I was called in and found this little patient with a pulse of only 42, and very depressed, and the parents still administering veratrum viride, muriate of ammonia, and aromatic spirits of ammonia, under the direction of the attending M. D. I immediately stopped all medicine except antipyrine, grains 3, every hour for eight hours, and tr. opii camph., gtt. 3, every two hours. Patient commenced improving from the first dose.

Case 5.—Henry F——, æt twenty-one. Lobular pneumonia. Temperature, 103°; pulse, 100; respiration, 32. Administered antipyrine, grains 10, every three hours. Discharged from my care, cured, in ten days.

Case 6.—Elmer D——, æt ten. Lobular pneumonia. Temperature, 102°; pulse, 96; respiration, 28. Administered antipyrine, grains 10, every four hours. Discharged him, cured, in ten days.

In these cases profuse perspiration followed the administration of the antipyrine, and the respiration and pulse were immediately depressed to nearly normal, while the temperature fell to 99° or 100°.

The only other treatment was the administration of a cathartic, and the application of hot fomentations to the lungs.

I would like to hear from others upon this subject; also upon the propriety of giving ammonia muriate in the active inflammatory stage of broncho-pneumonia.

**The Influence of Antipyrine on the Temperture and Tissue Change in Children.**—Dr. Jacubovitsch, of the Children's Clinic, Academy of St. Petersburgh, concludes a treatise on this subject as follows:

1. Antipyrin lowers the temperature as well in healthy as in sick children, but in the case of the former it sinks less than in the latter.

2. The force of the depression does not invariably depend upon the amount of the dose.

3. In children idiosyncrasy has an influence upon the fall of temperature outside of the mere largeness of dose, for very large doses sometimes have no effect.

4. Under the action of very large doses the temperature never continues at low figures longer than twenty hours.

5. The greatest depression is observed at midnight, and then the temperature gradually rises.

6. In the case of healthy children the temperature cannot be so much depressed as in those suffering from fever.

7. Small children can, in most cases, bear large doses for from one to two days well. Vomiting is rare, collapse or convulsions are not observed ; sweating is not constant.

8. Electro-muscular irritability rises with children from the use of the alkaloid, which can be explained by the experiments of Devenne upon frogs and puppies, through irritation of the musculo-motor centres.

9. In a small number of cases the daily quantity of urine is increased, and the specific gravity lowered ; but in the majority of cases the quantity of urine is diminished by a half or more, is much concentrated, syrup-like, with increased specific gravity ; the discharge of urine is notably hindered.

10. Forty-eight hours after the last dose the daily quantity of all urinary constituents surpasses that of the day before the exhibition of the alkaloid.

11. By means of chloride of iron and iodide of potassium, antipyrin can be discovered in the urine not later than forty-eight hours after the last dose.

In conclusion, we must say that, notwithstanding the facts observed by us, the question of the use of antipyrin in fever can not be answered with a positive opinion. It seems to us, however, that there can remain no doubt that, notwithstanding its great antipyretic power, a long-continued exhibition of the medicine to the same patient would be impossible if our exhibit of the retention of oxidation products should be confirmed.—*Am. Prac. and News.*

**Embalming** —The following formula will preserve the body if the injection is properly done : Take of the solution of chloride of zinc (U. S. Ph.), 1 gallon ; solution of chloride of sodium, 6 ounces to the pint of water, 6 pints ; solution of bichloride of mercury, 1 ounce to the pint of water, 4 pints ; alcohol, 4 pints ; carbolic acid (pure), ½ pint ; carbolic acid dissolved in glycerin, 1½ pints. Mix all the ingredients, and a clear solution of 3 gallons results, which is the proper amount for a body weighing 150 pounds. The solution may be injected into the aorta, but it is much less trouble to inject into the brachial or femoral artery, or the femoral vein may be selected. If an artery is used, the injection should be towards the capillaries, and if a vein, towards the heart. To satisfactorily inject a subject, a good anatomical syringe is desirable, but a gravity syringe can be improvised with rubber tubing, a stop-cock, and a terminal glass tube with the tip drawn to a fine point. I would suggest to Dr. Kennedy that he experiment on a few animals, and then he can devise a formula to suit himself. I have found that a fluidrachm of the solution recommended is sufficient for each ounce of weight of the animal to be preserved. For preserving human bodies, 2½ fluid ounces for each pound is a safe estimate.—DR. H. R. TILTON, Surgeon U. S. A., in *New York Medical Record.*

**Antipyrin in Typhoid Fever.**—A dose of 25 grains for an adult, repeated in an hour if the temperature has not begun its downward course, will accomplish all we require for the safety and wellbeing of our patient. Bring the temperature to 101° or 102° for four or five hours, and the pernicous effect of a continuous hyperpyrexia on the heart will be stayed. The later stages of the disease will afford valuable evidence of the antipyretic effect, inasmuch as the heart will respond to stimulants when it begins to flag, during the third or fourth week.

Dr. Baruch summarizes his conclusions as follows: "A proper use of antipyretics modifies the course of typhoid fever, and diminishes the mortality, because it diminishes the chief danger—heart-failure—and because it enables us to render the patient more comfortable, and therefore more quiet and tractable."—*Medical Age.*

**Strangulated Hernia Treated by Puncture.**—S. M——, aged sixty-three, a thin, flabby, white-faced, average-size man; had always enjoyed tolerably good health, with the exception of hernia, which had troubled him for several years. Latterly it had been coming down more frequently, increasing in size, and becoming more difficult to reduce.

On August 7th he was seized with a sharp attack of diarrhœa. Next morning, about 8 o'clock, while straining at stool, the hernia suddenly appeared in the left scrotum. All the means of reduction which had formerly been successful were tried, but failed. I saw him about 11 o'clock. The tumor was red and glistening, and reached half-way down to the knee. Symptoms of strangulation were well marked. The ordinary manipulation was used, but to no purpose; indeed, it was not long continued, as it gave rise to intense pain. Morphine was injected below the skin, frequent enemata were given, and bags of ice applied. After two hours, the symptoms continuing unabated, taxis, under chloroform, was again tried, but with no better result. As a large part of the tumor was tympanitic, evidently consisting of intestine distended with flatus, it occurred to me that, were this removed, reduction would be facilitated. I accordingly introduced a small trocar and cannula, and freed a large quantity of flatus. The hernia was reduced to a third of its original size, and its contents, without the slightest difficulty, returned into the abdominal cavity. The patient slowly fell asleep, and slept for several hours. Next day he was comparatively well, was kept still, and fed on milk diet. The bowels opened naturally that night, and on the third day he was out of bed, and going about the house. His recovery has been uninterrupted.

Whether this method has been used before, or not, I do not know, but, at any rate, in this case it was eminently successful.—*British Medical Journal.*

**Before attempting to remove foreign bodies** from any of the cavities of the body which are covered with mucous membrane, where possible, always thoroughly anæsthetize the part with a solution of cocaine. This not only relieves the subsequent manipulations of pain, but the effect of cocaine upon the mucous membrane, even when the latter is hypertrophied, is to greatly relax it and to retract it upon the tissues which it covers. Mr. Bryson Delevan has already applied the knowledge of this fact to the removal of foreign bodies from the nasal cavity, and Dr. E. J. Moure, of Bordeaux, has made use of it in catheterization and insufflation of the eustachian tubes. In a note to *La France Medicale*, Dr. Moure states that in those cases of subacute inflammation of the tubes where the membrane is covered with a thick, viscid, catarrhal exudation which prevents the admission of air to the middle ear, resisting all attempts at insufflation by ordinary means, he finds no difficulty whatever after the application of a ten per cent. solution of cocaine. This is applied first to the nasal conduit, being carried a few moments later to the level of the naso-pharyngeal cavity, and thence, after the lapse of a few moments more, as far as possible into the tubes.—*St. Louis Medical Journal.*

**Perchloride of Iron in Anthrax.**—At a meeting of the Lucerne Medical Society, Dr. Alfred Steiger made a communication on the local use of perchloride of iron in anthrax (as recommended by an English practitioner of Buenos Ayres, whose name could not be ascertained). Seven patients treated by the author all recovered within a few days after a single painting with liquor ferri sesquichloridi. "Perchloride of iron is," he said, "a true specific for anthrax; even advanced cases are cut short by it." He obtained also excellent results from the same drug in many cases of ingrowing nails. After cleansing the ulcerated surface he painted it twice daily with a fine brush, and ordered rest. Within a few days the soft parts became hard and insensitive and contracted, while the edge of the nail was raised from its groove. The internal administration of the drug gave very good results in Werlhof's disease, albuminuria after scarlatina, diabetes melitus, and renal and vesical hematuria.—*British Medical Journal.*

**The Proper Use of Antipyrine.**—Pavay has employed this drug in a large number of cases, and give some useful rules for its administration. He adopts a middle course in regard to dosage. When the temperature does not exceed 103° F., he divides 31 grs. into three powders and administers one powder every half hour. If the thermometer register 104°, three doses are given, as before, each consisting of 15½ grains. With a temperature of 105° and above, he gives 62 grains in four doses, half an hour apart. It is seldom, the writer asserts, that the temperature fails to fall from 2–4°, and to remain lowered from six to sixteen hours. If for any reason the stomach will not retain the drug, it may be given by the rectum in doses of from 30–45 grains, or hypodermically in a fifty per cent. solution.—*New York Medical Journal.*

"**Black Eye.**"—There is nothing to compare with the tincture or a strong infusion of *capsicum annuum* mixed with an equal bulk of mucilage of gum-arabic and with the addition of a few drops of glycerine. This should be painted all over the bruised surface with a camel's-hair pencil and allowed to dry on, a second or third coating being applied as soon as the first is dry. If done immediately after the injury is inflicted, this treatment will almost invariably prevent the blackening of the bruised tissue. The same remedy has no equal in rheumatic, sore, or stiff neck.—*St. Louis Medical and Surgical Journal.*

**Fracture of the Patella.**—Dr. Francis J. Shephard, in *Canada Medical and Surgical Journal*, says: For several years past I have adopted Mr. Heath's method of treatment by aspiration, if there is effusion; and then the application of a plaster of Paris bandage. The results obtained are good, and in the last two cases, in which the plaster was applied within an hour of the receipt of the injury, the union after several months was particularly close. The method had these advantages: it is safe simple, and can be applied by medical men in the country (who have not the appliances of a hospital at hand) with the greatest ease. The materials are always ready, and take but little time and skill to apply.

**Heller's Albumin Test.**—Dr. Thomas K. Morton, Philadelphia (*Medical News*), May 8, 1886, says take a very small test-tube, fill it one-third with nitric acid; then fold filter paper so as to make a funnel, and insert its point into the mouth of the test-tube; then pour into the funnel about a drachm of the suspected urine, which will slowly filter through the paper and run down perfectly clear upon the acid, and, should albumin be present, show it as a sharp-cut white ring at the junction of the two liquids.

**Treatment of Ingrowing Toe-Nail.**—A Dr. Miall, writing in the *British Medical Journal*, claims to cure cases of this trouble without rest, by the use of tannin. A concentrated solution (an ounce of perfectly fresh tannic acid, dissoved in six drams of pure water, with a gentle heat) must be painted on the soft parts twice a day. After the first application the patients had no pain or lameness, and went about their work immediately. In about three weeks the cases were cured.—*Southern Cliuic*

**The** applications of electricity become more varied every day. Telebarometers, telethermometers, telemanometers and telehydrobarometers have recently been invented, which respectively record, at distant points, air-pressure, heat, steam-pressure and water stages. A California electrician has also invented a process whereby gold, silver and copper can be instantly smelted by a lightning stroke.—*Electrical World.*

**Rival to Cocaine.**—A rival of cocaine is described in an Australian medical journal. It is an alkaloid derived from the *Euphorbia Drummondii*, a plant which grows plentifully in the neighborhood of Port Germain, Australia; and Dr. Reid of that town gives the new local anæsthetic the name of "drumine." Dr. Reid believes this drug will be found a valuable adjuvant in the treatment of many painful affections.—*Popular Science News.*

**Bi-Tartrate of Potash in Pregnancy.**—Dr. E. Anderson writes (in *Maryland Medical Journal*): My experience has been, that if the bi-tartrate of potash be administered to a pregnant woman for one month pior to confinement, in a sufficient amount to bring about free action of the kidneys and bowels, puerperal convulsions will never occur.

